# Accurate breast cancer diagnosis using a stable feature ranking algorithm

**DOI:** 10.1186/s12911-023-02142-2

**Published:** 2023-04-06

**Authors:** Shaode Yu, Mingxue Jin, Tianhang Wen, Linlin Zhao, Xuechao Zou, Xiaokun Liang, Yaoqin Xie, Wanlong Pan, Chenghao Piao

**Affiliations:** 1 School of Information and Communication Engineering, Communication University of China, Beijing, China; 2 Department of Radiology, The Second Affiliated Hospital of Shenyang Medical College, Shenyang, China; 3 Shenzhen Institute of Advanced Technology, Chinese Academy of Sciences, Shenzhen, China; 4 Experimental Teaching Center for Pathogen Biology and Immunology, North Sichuan Medical College, Nanchong, China

**Keywords:** Breast cancer diagnosis, Feature ranking stability, Machine learning, Decision making

## Abstract

**Background:**

Breast cancer (BC) is one of the most common cancers among women. Since diverse features can be collected, how to stably select the powerful ones for accurate BC diagnosis remains challenging.

**Methods:**

A hybrid framework is designed for successively investigating both feature ranking (FR) stability and cancer diagnosis effectiveness. Specifically, on 4 BC datasets (BCDR-F03, WDBC, GSE10810 and GSE15852), the stability of 23 FR algorithms is evaluated via an advanced estimator (*S*), and the predictive power of the stable feature ranks is further tested by using different machine learning classifiers.

**Results:**

Experimental results identify 3 algorithms achieving good stability ($S \ge 0.55$) on the four datasets and generalized Fisher score (GFS) leading to state-of-the-art performance. Moreover, GFS ranks suggest that shape features are crucial in BC image analysis (BCDR-F03 and WDBC) and that using a few genes can well differentiate benign and malignant tumor cases (GSE10810 and GSE15852).

**Conclusions:**

The proposed framework recognizes a stable FR algorithm for accurate BC diagnosis. Stable and effective features could deepen the understanding of BC diagnosis and related decision-making applications.

## Background

Breast cancer (BC) is one of the most frequently diagnosed cancers among women worldwide. In 2020, it caused 2.26 million new cases and 0.68 million deaths [1]. As a transitioning country, China is facing a growing burden, since the number of new cases is near 0.42 million [2]. Much worse is transitioning countries have lower incidence rates but much higher death rates than transitioned countries [1]. The substantial BC burden in developing and low-resource countries calls for cost-effective screening and diagnostic services to improve survival rates and quality of life [3].

Many techniques have been developed for BC screening and diagnosis [4, 5]. Mammography (MAM) is the gold standard for BC screening. Due to high-resolution imaging of internal anatomy, it benefits the observation of suspicious lesions. To make a diagnosis of cancer, fine needle aspiration (FNA) biopsy test is needed. It obtains a sample of breast lump cells, and a pathologist checks whether the sample contains any cancer cells [4]. Gene expression profiling tests analyze genes within cancer cells and can help decide whether a patient is expected to benefit from additional treatment after surgery [5]. Some other modalities, such as ultrasound tomography [6], are under pre-clinical trial for investigating BC diagnosis and prognosis.

Computer-aided diagnosis (CAD) models have also been built to facilitate BC diagnosis [7, 8]. A CAD model consists of feature extraction, feature selection, and malignancy prediction. Feature extraction is to design or collect variables or predictors for breast tumor representation. The features can be computed from intensity analysis, shape description and texture quantification [7]. Since the feature dimensionality grows dramatically, feature selection becomes increasingly important, and its purpose is to find a subset of features by removing redundant and irrelevant ones [9]. According to the output type, feature selection methods can be categorized into feature ranking (FR) and subset feature selection (SFS) groups [9]. To differentiate benign and malignant cases, popular classifiers not limited to artificial neural network (ANN), *K*-nearest neighbors (KNN), linear discriminant analysis (LDA), naive Bayes (NB), random forest (RF) and support vector machine (SVM) are used [10]. Recently, deep learning has updated CAD performance [11]. It fuses feature extraction, feature selection and cancer prediction into a seamless optimization procedure [12]. Novel architectures have been designed, and technical strategies have also been suggested [8].

However, two shortcomings are observed in the understanding of FR/SFS algorithms for decision-making applications. Firstly, the stability has rarely been studied. Specifically, few of FR/SFS algorithms are evaluated [13–16], and stability estimators are not yet comprehensive [17]. Secondly, the superiority of an FR/SFS algorithm is overwhelmingly determined by its predictive power and thus, performance-oriented. The underestimation of stability decreases user confidence and hampers the deployment of FR/SFS algorithms in real-world applications.

To well address the above-mentioned shortcomings, a hybrid framework is proposed for investigating both FR/SFS stability and diagnosis effectiveness. To the best of our knowledge, this is the first work devoted to evaluating the stability and effectiveness of more than twenty FR algorithms on BC data analysis (BCDR-F03, WDBC, GSE10810 and GSE15852). The contributions of this work can be summarized as follows:

1. A hybrid framework is designed in which both the FR/SFS stability and the diagnosis effectiveness can be evaluated successively.
2. The stability of 23 FR algorithms is assessed on 4 BC datasets via an advanced estimator, and 3 FR algorithms are identified stable.
3. The predictive power of stable ranks is tested, and generalized Fisher score (GFS) leads to superior performance regardless of classifiers.
4. GFS ranks suggest shape features are vital in image analysis (BCDR-F03 and WDBC) and using a few of genes can well differentiate malignant cases from benign ones (GSE10810 and GSE15852).

## Related work

**Fig. 1:**
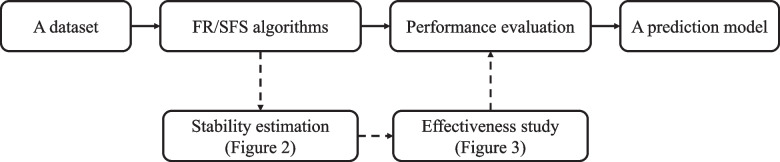
The performance-oriented (solid-line arrows) and the proposed stability-first FR/SFS (dashed-line arrows) frameworks for building a CAD model. (The figure can be enlarged for viewing)

In most studies, the superiority of FR/SFS algorithms is defined by the predictive power as shown with solid-line arrows in Fig. 1. For instance, performance comparison of SFS methods and classifiers on glioma grading is quantified by using the balanced accuracy and the area under the curve [10], and FR outcomes followed by classifiers are evaluated using precision, sensitivity and F-measure for finding the most significant features [18].

Few studies have explored FR/SFS stability and predictive power at the same time. For BC risk forecasting, 6 methods are assessed via correlation coefficient and Jaccard index [13]. For colorectal cancer prediction, 6 methods are evaluated using 3 similarity-based measures [14]. On gene datasets, 6 methods are analyzed with 3 estimators [15]. And on small-sample data analysis, relative weighted consistency, partially adjusted average Tanimoto index and correlation-based similarity measures are used [16].

However, these studies [13–16] are not comprehensive, because the estimators used lack one or more properties a good estimator should possess [17], and subsequently, the conclusions might be untenable. Therefore, building a hybrid framework to investigate both stability and effectiveness of FR/SFS algorithms is meaningful. Table 1 shows from the number of FR/SFS algorithms and estimators involved and from whether the estimators satisfy the full propertiers of a good estimator [17].

**Table 1:** Comparison of related works

	FR/SFS	estimators	satisfied
[13]	6	2	no
[14]	6	3	no
[15]	6	3	no
[16]	1	3	no
Ours	23	1	yes

This study differs from the previous studies [13–16]. Overall, 23 algorithms are evaluated, surpassing that of each previous study. Meanwhile, an advanced estimator [17] is used, and the dynamic change of FR stability is quantified regarding the number of selected features. Moreover, on 4 BC datasets, stable algorithms are identified, and their effectiveness is assessed on malignancy prediction of breast tumor cases. In addition, selected features are analyzed as potential BC signatures by literature screening, and the findings may pave the way for understanding the disease occurrence and diagnosis.

## Materials and methods

In this section, data collection, FR algorithms, stability estimator, machine learning classifiers and experimental design are described. To preserve the readability, major notations are summarized in Table 2.

**Table 2:** Main notations

**Notation**	**Description**
*S*	the value of a stability estimator
*K*	the number of nearest neighbors
(*X*, *y*)	a sample of features (*X*) and its label *y*
$f_{i,k}$	the index of the $k^{th}$ feature after the $i^{th}$ ranking
*m*	the number of features in stability analysis
*N*	the number of feature ranking experiments
*n*	the number of features used for BC diagnosis
*M*	the number of BC diagnosis experiments

### Data collection

Four datasets are analyzed. BCRD-F03 [19] includes 406 breast lesions (230 benign and 176 malignant) and 736 MAM images. For lesion representation, 17 features are derived from intensity analysis (mean, median, standard error, maximum, minimum, kurtosis, and skewness), shape description (area, perimeter, x-center, y-center, circularity, elongation, and form) and texture quantification (contrast, correlation, and entropy). To avoid one lesion with multiple images, the first feature record of each lesion is used.

Wisconsin Diagnostic Breast Cancer (WDBC) [20] contains 357 benign and 212 malignant instances. For a FNA image, 10 features (radius, texture, perimeter, area, smoothness, compactness, concavity, concave points, symmetry, and fractal dimension) are computed. Besides mean values, the standard error and the “worst” (or largest) values of features are collected.

Other datasets are from the Gene Expression Omnibus (GEO) [21]. GSE10810 comprises 31 tumor samples and 27 control samples of breast specimens, and 18,382 gene profiles are provided for each sample [22]. GSE15852 provides 43 tumor samples and 43 control samples of Malaysian women, and 22,283 gene expression data points are collected [23].

Table 3 shows the dataset information. The goal is to recognize malignant samples from benign ones by using medical images (BCDR-F03 and WDBC), or gene profiles (GSE10810 and GSE15852).

**Table 3:** Summary of the datasets used in this study

	benign (training/testing)	malignant (training/testing)	feature number (*p*)	source
BCDR-F03	230 (141/89)	176 (141/35)	17	MAM
WDBC	357 (170/187)	212 (170/42)	30	FNA
GSE10810	27 (22/5)	31 (22/9)	18382	gene
GSE15852	43 (34/9)	43 (34/9)	22283	gene

### FR algorithms

Twenty-three methods in the matFR toolbox [24] are evaluated, and the other methods beyond time expectation ($\ge 0.5$ hour per iteration) on GSE15852 are discarded. In general, the core ideas of used algorithms are based on absolute values of *t*-test [25], relative entropy [26], Bhattacharyya distance [27], the area between the empirical receiver operating characteristic curve and random classifier slope [28], absolute values of Mann-Whitney test [29], ReliefF [30], the least absolute shrinkage and selection operator [31], correlation analysis [32], generalized Fisher score (GFS) [33], Gini score [34], Kruskal-Wallis test [35], pairwise feature proximity (PWFP) [36], min-max local structure information [37], local learning-based clustering [38], eigenvector centrality [39], probabilistic latent graph-based measure space [40], concave minimization and SVM [41], the convergence properties of the power series of matrices [42], Laplacian score [43], L$_{2,0}$-norm equality constraints (LNEC) [44], adaptive structure learning [45], robust spectral learning of the spectrum information of the graph Laplacian [46], and L$_{2,1}$-norm minimization on processes of both label learning and feature learning [47]. Full details of the algorithms can be found in the original publications.

### Stability estimator

The stability estimator proposed in [17] is used, and it possesses the full properties a good estimator should hold. It recasts the procedure of FR/SFS stability measure as the estimation of a random variable, and corresponding population parameters are explicitly embedded. After the sampling distribution is identified, tools are provided to estimate the confidence intervals and to perform the hypothesis tests. Importantly, the estimator allows for reliable comparison across different FR/SFS procedures.

In addition, stability values (*S*) above 0.75 represent excellent agreement of feature sets beyond chance, the values below 0.40 reveal poor agreement between sampled feature sets, and the values in the range of 0.40 and 0.75 indicate intermediate to good agreement.

### Machine learning classifiers

To avoid potential over-fitting, simple classifiers are used. ANN is with one hidden layer (ANN01) and with two hidden layers (ANN02), and 10 neurons are embedded in each hidden layer. KNN is a nonparametric classifier, and a new instance is grouped based on the class labels of the majority of *K* nearest neighbors. LDA is to find a linear combination of features for separating new instances. NB is a probabilistic classifier based on the basic Bayes theorem with an independence assumption between features and class labels. Linear SVM is a supervised learning classifier and groups new instances into different classes by using optimized hyper-planes.

### Evaluation metrics

To quantify the prediction performance, area under the receiver operating characteristic curve (AUC), accuracy (ACC), sensitivity (SEN), specificity (SPE), negative predictive value (NPV), F-measure and Matthews correlation coefficient (MCC) are used.

The metrics have been widely used in binary classification problems ($y \in \{0, 1\}$), and higher values indicate better prediction results. In this study, the label of a benign case is $y=0$, and the label of a malignant case is $y=1$.

### Experimental design

#### Estimation of FR stability

**Fig. 2:**
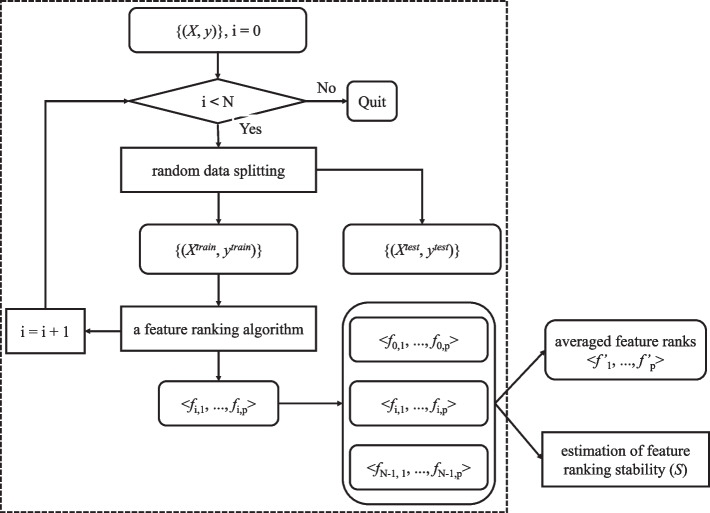
The procedure of FR stability estimation. On a given dataset, *N* iterations of feature ranking are conducted that yield average feature ranks and stability values *S*. (The figure can be enlarged for viewing)

Figure 2 shows how to estimate FR stability. In each iteration, a dataset $\{(X, y)\}$ is divided into a training set $\{(X^{train}, y^{train})\}$ and a testing set $\{(X^{test}, y^{test})\}$, and each FR method yields a feature rank on the training set. Moreover, $<f_{i,1}, ..., f_{i,k}, ..., f_{i,p}>$ is the output of the $i^{th}$ running of the *p* features, and $f_{i,k}$ is the ranking index of the $k^{th}$ feature. Finally, feature ranks are averaged as an output.

The other output is the stability value (*S*) when top-*m* features are selected. Notably, an FR algorithm generates a feature rank in a descending order in terms of feature importance. When the number (*m*) of features is defined, it yields a subset of features. In this study, $N=30$, and *m* ranges from 3 to 10 with equal interval of 1. Specifically, when $m = 3$ and $S \ge 0.55$, an FR algorithm is assumed to be stable on the dataset.

As shown in Table 3, when a dataset is divided into two subsets, the number of benign and malignant cases is set equal in the training set ($\approx$ 80% of the group with fewer cases).

#### Effectiveness of feature ranks on BC diagnosis

**Fig. 3:**
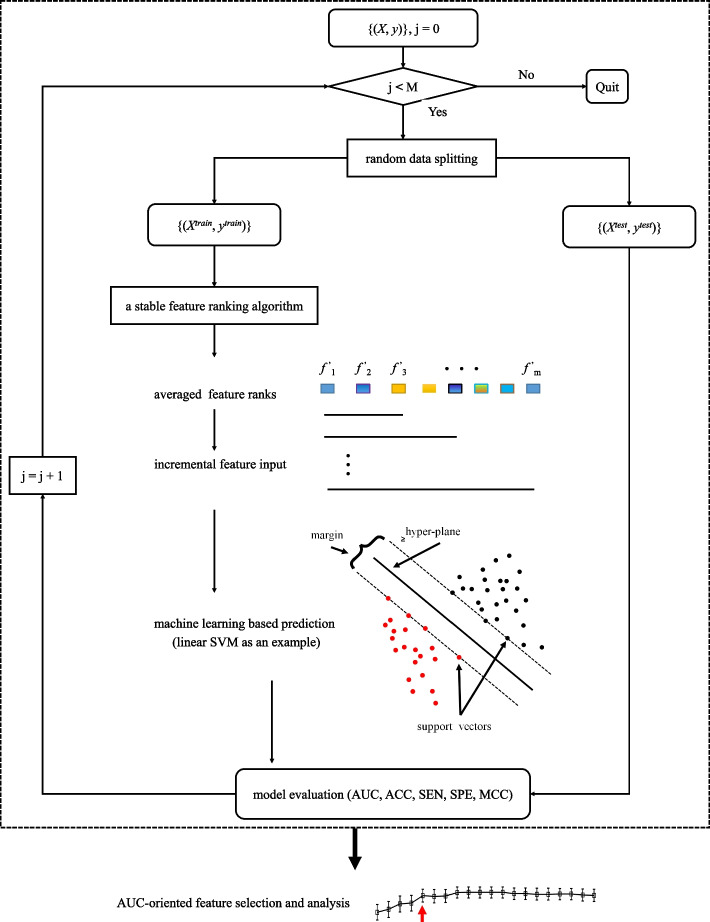
The procedure of estimating the effectiveness of feature ranks on BC diagnosis. On a given dataset, top-*m* features are incrementally added and *M* iterations of data splitting are conducted for machine learning based malignancy prediction. (The figure can be enlarged for viewing)

For a stable FR algorithm, its *N*-times of feature ranks are averaged, and then, the predictive power of top-*m* features is explored on BC diagnosis. Figure 3 shows the procedure, the value of *m* increases incrementally, and linear SVM is an example of classifiers.

After a model is trained, the optimal number (*n*) of features is determined by balancing model complexity and prediction performance, *i*.*e*. feature number vs. AUC value, on the testing set. Notably, $M=50$, the feature number progresses from 1 to 10, and the prediction results and selected features are analyzed.

### Statistical analysis

The Wilcoxon rank sum test is used to analyze the values of evaluation metrics. It is non-parametric for testing two populations with independent samples. The *p*-value less than 0.05 is defined as the significance level to reject the null hypothesis of equal medians between two distributions.

### Implementation and platform

The proposed framework runs on a Win10 system (one Intel (R) Core (TM) i9-11980HK CPU (3.20 GHz), and 64.0 GB RAM). Algorithms are implemented with MATLAB R2018a (MathWorks, Natick, MA, United States). FR algorithms are from matFR^1^, the stability estimator is from github^2^, and classifiers and statistical analysis are implemented with embedded functions. In addition, except for $K = 5$ (KNN), the other parameters of FR methods, estimator, classifiers and Wilcoxon rank sum test are set to default values.

## Results

### Estimated FR stability

Stability values are shown in Tables 4 and 5, and values with $S \ge 0.55$ when $m=3$ are in red. Table 4 shows most algorithms achieving good stability (BCDR-F03 and WDBC). The *S* values of [25, 35, 36, 39, 43, 46, 47] are near or larger than 0.80 on both datasets. On contrast, [32, 37] on BCDR-F03 and [32, 41] on WDBC are highly sensitive to data perturbation.

**Table 4:** Estimated FR stability values on medical image datasets (BCDR-F03 and WDBC)

		top-3	top-4	top-5	top-6	top-7	top-8	top-9	top-10
BCDR-F03	[25]		0.85	0.77	0.76	0.77	0.78	0.74	0.71
	[26]		0.81	0.80	0.69	0.66	0.68	0.64	0.63
	[27]		0.82	0.80	0.68	0.66	0.67	0.63	0.63
	[28]		0.79	0.89	0.79	0.73	0.71	0.71	0.79
	[29]		0.76	0.84	0.80	0.82	0.93	0.84	0.78
	[30]	0.46	0.39	0.37	0.39	0.39	0.40	0.39	0.36
	[31]		0.59	0.54	0.50	0.46	0.43	0.47	0.47
	[32]	0.06	0.08	0.07	0.10	0.11	0.14	0.14	0.12
	[33]		0.74	0.77	0.83	0.84	0.80	0.86	0.80
	[34]		0.82	0.77	0.73	0.63	0.59	0.59	0.61
	[35]		0.81	1.00	1.00	1.00	1.00	1.00	1.00
	[36]		0.89	0.90	0.91	0.92	0.92	0.99	0.90
	[37]	0.24	0.27	0.35	0.36	0.35	0.36	0.38	0.36
	[38]		0.89	0.85	0.91	1.00	0.92	0.91	0.80
	[39]		1.00	0.88	0.75	0.70	0.73	0.73	0.79
	[40]	0.52	0.62	0.63	0.62	0.62	0.54	0.48	0.44
	[41]		0.82	0.79	0.84	0.75	0.68	0.71	0.71
	[42]		1.00	0.87	0.78	0.78	0.79	0.83	0.80
	[43]		1.00	0.93	0.89	1.00	0.99	0.92	0.88
	[44]		0.80	0.94	0.85	0.99	0.88	0.88	0.99
	[45]		0.80	0.82	0.78	0.82	0.79	0.69	0.66
	[46]		0.90	0.93	0.94	0.87	0.91	0.89	0.86
	[47]		0.85	1.00	1.00	1.00	0.96	0.89	1.00
WDBC	[25]		1.00	1.00	0.95	0.92	0.89	0.92	1.00
	[26]		0.76	0.78	0.86	0.88	0.86	0.85	0.88
	[27]		0.71	0.84	1.00	0.88	0.95	0.94	0.92
	[28]		0.87	1.00	0.94	0.90	0.89	0.97	0.93
	[29]		1.00	0.88	0.86	0.80	0.86	0.91	1.00
	[30]		0.59	0.56	0.53	0.53	0.51	0.50	0.48
	[31]		0.63	0.57	0.54	0.50	0.47	0.47	0.45
	[32]	0.08	0.12	0.14	0.17	0.21	0.22	0.25	0.27
	[33]		0.98	0.97	0.91	0.92	0.95	0.97	1.00
	[34]		0.78	1.00	0.88	0.82	0.85	0.88	0.90
	[35]		0.97	1.00	0.89	1.00	0.94	1.00	0.96
	[36]		1.00	1.00	1.00	1.00	1.00	1.00	0.92
	[37]		0.95	0.96	1.00	0.91	1.00	0.98	0.90
	[38]		0.86	0.90	0.83	0.80	0.78	0.80	0.84
	[39]		1.00	0.88	0.99	1.00	0.96	0.98	0.98
	[40]		0.97	0.91	0.85	0.87	0.95	0.93	0.99
	[41]	0.19	0.22	0.23	0.23	0.23	0.23	0.25	0.26
	[42]		1.00	0.88	0.98	1.00	0.96	0.97	0.98
	[43]		0.87	1.00	0.98	1.00	0.98	0.95	0.98
	[44]		1.00	0.90	0.90	0.96	1.00	1.00	0.98
	[45]	0.47	0.57	0.66	0.72	0.77	0.81	0.83	0.88
	[46]		0.87	1.00	1.00	1.00	0.98	0.92	1.00
	[47]		0.86	0.77	0.75	0.73	0.80	0.82	0.86

Values with $S \geq 0.55$ when $m = 3$ are highlighted with red color

Table 5 shows several algorithms with good stability on the gene datasets. On GSE10810, [27, 33, 36, 41, 44] are stable with $0.58 \le S \le 0.78$. On GSE15852, [33, 35, 36, 39, 42–44] have *S* values within [0.56, 0.85]. Notably, *S* values of some algorithms are close to zero, such as [32, 38, 40, 45] on GSE10810 and [32, 40, 45] on GSE15852.

**Table 5:** Estimated FR stability values on gene expression datasets (GSE10810 and GSE15852)

		top-3	top-4	top-5	top-6	top-7	top-8	top-9	top-10
GSE10810	[25]	0.44	0.55	0.54	0.52	0.52	0.51	0.50	0.50
	[26]		0.87	0.81	0.77	0.73	0.71	0.72	0.73
	[27]	0.44	0.52	0.52	0.49	0.49	0.50	0.50	0.49
	[28]	0.52	0.51	0.46	0.44	0.43	0.44	0.47	0.47
	[29]	0.21	0.26	0.25	0.25	0.25	0.25	0.25	0.26
	[30]	0.43	0.42	0.41	0.41	0.41	0.41	0.42	0.41
	[31]	0.15	0.20	0.20	0.20	0.21	0.23	0.24	0.24
	[32]	0.00	0.00	0.00	0.00	0.00	0.00	0.00	0.00
	[33]		0.50	0.49	0.48	0.48	0.47	0.49	0.48
	[34]	0.52	0.51	0.46	0.44	0.43	0.44	0.47	0.47
	[35]	0.31	0.40	0.39	0.39	0.37	0.39	0.39	0.41
	[36]		0.77	0.82	0.85	0.86	0.84	0.84	0.85
	[37]	0.11	0.12	0.13	0.16	0.18	0.21	0.22	0.24
	[38]	0.00	0.00	0.01	0.01	0.01	0.01	0.01	0.01
	[39]	0.46	0.51	0.59	0.66	0.73	0.78	0.80	0.82
	[40]	0.00	0.00	0.00	0.00	0.00	0.00	0.01	0.01
	[41]		0.56	0.52	0.50	0.46	0.45	0.44	0.43
	[42]	0.46	0.50	0.59	0.66	0.71	0.78	0.80	0.81
	[43]	0.25	0.28	0.30	0.30	0.33	0.35	0.36	0.37
	[44]		0.64	0.64	0.65	0.67	0.66	0.66	0.67
	[45]	0.05	0.05	0.07	0.09	0.09	0.09	0.09	0.10
	[46]	0.51	0.52	0.50	0.48	0.51	0.54	0.57	0.59
	[47]	0.17	0.18	0.18	0.21	0.22	0.23	0.24	0.26
GSE15852	[25]	0.40	0.49	0.51	0.52	0.59	0.61	0.61	0.61
	[26]	0.51	0.51	0.56	0.60	0.61	0.60	0.62	0.63
	[27]	0.47	0.51	0.57	0.59	0.58	0.57	0.57	0.58
	[28]	0.28	0.33	0.38	0.40	0.40	0.42	0.44	0.47
	[29]	0.48	0.52	0.59	0.65	0.70	0.73	0.76	0.76
	[30]	0.31	0.41	0.46	0.52	0.55	0.58	0.60	0.61
	[31]	0.16	0.21	0.21	0.20	0.20	0.21	0.21	0.22
	[32]	0.00	0.00	0.00	0.00	0.00	0.00	0.00	0.00
	[33]		0.56	0.57	0.61	0.66	0.71	0.75	0.73
	[34]	0.21	0.24	0.26	0.30	0.32	0.35	0.38	0.40
	[35]		0.62	0.62	0.67	0.73	0.78	0.81	0.83
	[36]		0.88	0.88	0.86	0.88	0.88	0.89	0.90
	[37]	0.24	0.26	0.24	0.26	0.27	0.28	0.30	0.31
	[38]	0.53	0.46	0.44	0.42	0.43	0.42	0.43	0.42
	[39]		0.97	0.89	0.88	0.85	0.87	0.86	0.89
	[40]	0.00	0.00	0.00	0.00	0.00	0.00	0.00	0.00
	[41]	0.25	0.28	0.27	0.29	0.31	0.31	0.32	0.32
	[42]		0.96	0.89	0.89	0.86	0.86	0.88	0.89
	[43]		0.65	0.66	0.74	0.80	0.88	0.87	0.82
	[44]		0.54	0.56	0.57	0.59	0.60	0.61	0.63
	[45]	0.05	0.05	0.06	0.07	0.06	0.07	0.07	0.07
	[46]	0.54	0.58	0.56	0.55	0.55	0.55	0.55	0.55
	[47]	0.16	0.18	0.22	0.24	0.26	0.27	0.29	0.31

Values with $S \geq 0.55$ when $m = 3$ are highlighted with red color

According to the rule of $S \ge 0.55$ when $m=3$, there are 19, 20, 5, and 7 algorithms showing good stability on BCDR-F03, WDBC, GSE10810 and GSE15852, respectively. It is also found that PWFP is strongly stable ($\ge 0.70$) on all the datasets, LNEC obtains strong stability on BCDR-F03 and WDBC and good stability ($\ge 0.60$) on GSE10810, while the *S* values of GFS are relatively lower. Moreover, compared to *S* values on the gene datasets (GSE10810 and GSE15852), FR algorithms obtain much higher values on medical image datasets. Further observation reveals 3 FR algorithms (GFS [33], PWFP [36] and LNEC [44]) obtaining good stability on all the datasets, and the algorithms are focused on in follow-up analysis.

### Effectiveness of feature ranks on BC diagnosis

**Fig. 4:**
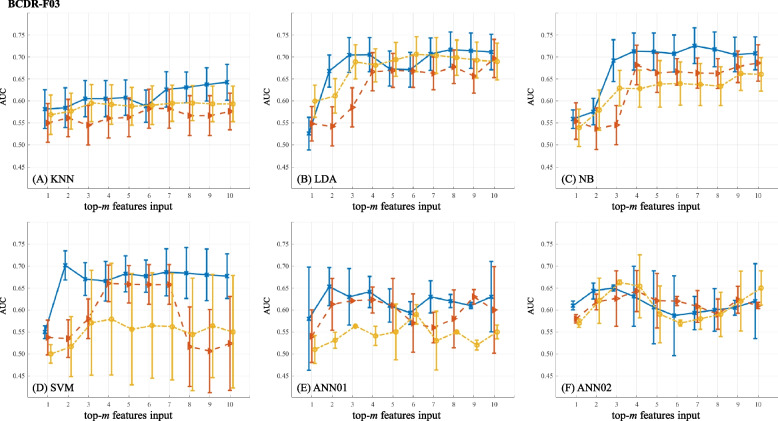
Predictive power of feature ranks on BCDR-F03. A plot shows the results of one classifier combined with different FR feature ranks. (The figure can be enlarged for viewing)

The predictive power of feature ranks on BC diagnosis is shown in Figs. 4 (BCDR-F03), 5 (WDBC), 6 (GSE10810) and 7 (GSE15852). In the figures, AUC values marked as solid lines with blue crosses, dashed lines with brown triangles and dot-dashed lines with yellow stars stand for the results from the ranks of GFS, PWFP and LNEC, respectively. In each plot, the horizontal axis denotes the number (*m*) of the features, and the vertical axis shows the AUC values using a specific machine learning classifier.

#### On the BCDR-F03

Figure 4 shows the prediction results on BCDR-F03 using different feature subsets. AUC values from GFS ranks are correspondingly larger than those from the other feature ranks on average. Based on GFS ranks, NB and SVM achieve better performance using 3 and 2 features, respectively. Meanwhile, using LDA as the classifier, LNEC leads close performance to GFS when 3 features are used. Out of the 17 image features, GFS prefers the “contrast”, “circularity” and “perimeter”.

#### On the WDBC

**Fig. 5:**
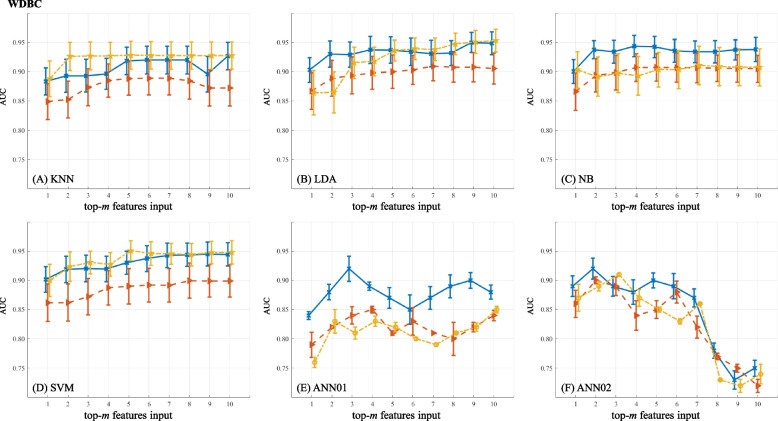
Predictive power of feature ranks on WDBC. A plot shows the results of one classifier combined with different FR feature ranks. (The figure can be enlarged for viewing)

Figure 5 shows AUC values when different feature subsets are used on WDBC. Comparatively, PWFP ranks cause worse results, and GFS and LNEC ranks lead to superior results. From the perspective of model simplicity, KNN with LNEC (2 features), LDA with GFS (2 features), NB with GFS (2 features), and SVM with GFS (2 features) or LNEC (2 features) achieve good results (AUC $\ge 0.90$). Out of the 30 image features, GFS ranks top of “the largest concave points”, “the largest perimeter” and “concave points”.

#### On the GSE10810

**Fig. 6:**
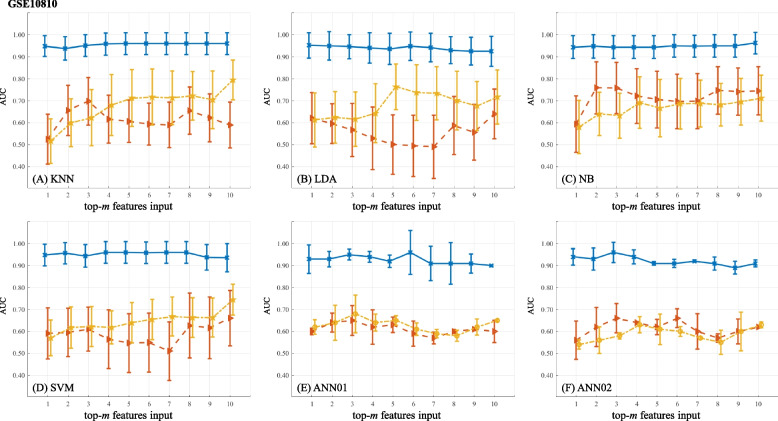
Predictive power of feature ranks on GSE10810. A plot shows the results of one classifier combined with different FR feature ranks. (The figure can be enlarged for viewing)

The change of AUC values along with selected features on GSE10810 is shown in Fig. 6. It suggests that GFS might identify a subset of discriminative features since the AUC values reach AUC $\approx$ 0.95 when few features are used. In addition, when classifiers change, the prediction performance remains good. In contrast, feature ranks from PWFP and LNEC cause poor AUC values ($\le$ 0.80). Out of 18,382 genes, GFS prefers “206930_at”, “243311_at” and “222083_at” as the most important ones.

#### On the GSE15852

**Fig. 7:**
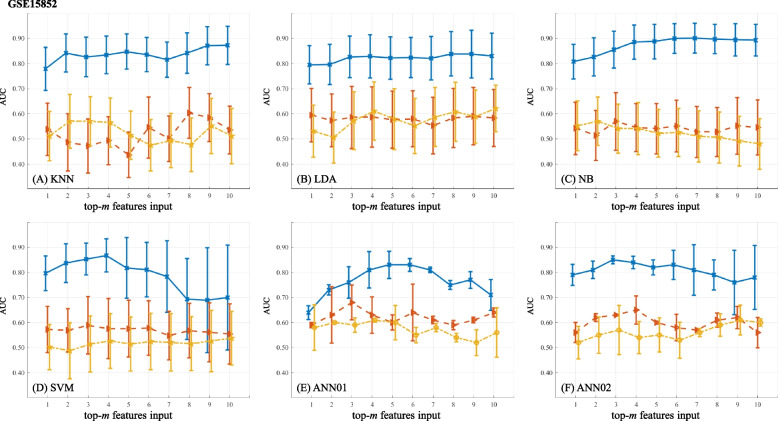
Predictive power of feature ranks on GSE15852. A plot shows the results of one classifier combined with different FR feature ranks. (The figure can be enlarged for viewing)

Figure 7 shows the AUC values on GSE15852. Again, AUC values from GFS ranks are much better than those from the other two feature ranks, and using 2 to 4 features leads to AUC $\ge$ 0.80. Out of the 22,283 gene profiles, GFS ranks top of the genes of “204997_at”, “210298_x_at” and “222317_at”.

#### Summary of the BC diagnostic performance

Table 6 presents the prediction results on the datasets when using different feature ranks, classifiers and selected features, and * denotes *p*-value < 0.05 of each metric within a feature rank. Generally, GFS ranks lead to higher metric values over the ranks from PWFP and LNEC when using same classifiers. Notably, the superiority of GFS ranks is observed from the gene data analysis with significantly better results (*p*-values $< 10^{-8}$) regardless of classifiers. In summary, for malignancy prediction, GFS ranks induce superior results when using 4 features as the NB input on BCDR-F03, using 2 features as the NB input on WDBC, using 2 genes as the SVM input on GSE10810 and using 4 genes as the NB input on GSE15852.

**Table 6:** Summary of BC diagnosis performance (* denotes *p*-value < 0.05 of one metric within a feature rank)

			*n*	AUC	ACC	SEN	SPE	NPV	F-measure	MCC
BCDR-F03	GFS	KNN	3	0.61±0.04	0.60±0.04	${\textbf {0.62}}\!\varvec{\pm }\!{\textbf {0.08*}}$	0.59±0.06	0.80±0.04	0.47±0.03	0.46±0.05
		LDA	3	0.70±0.04	0.75±0.03	0.59±0.08	0.82±0.02	${\textbf {0.84}}\!\varvec{\pm }\!{\textbf {0.05}}$	0.57±0.03	0.54±0.04
		NB	4	${\textbf {0.71}}\!\varvec{\pm }\!{\textbf {0.04}}$	0.77±0.03	0.59±0.10	0.84±0.05	0.84±0.07	${\textbf {0.59}}\!\varvec{\pm }\!{\textbf {0.03*}}$	${\textbf {0.58}}\!\varvec{\pm }\!{\textbf {0.03*}}$
		SVM	2	0.70±0.03	${\textbf {0.78}}\!\varvec{\pm }\!{\textbf {0.03}}$	0.53±0.07	${\textbf {0.87}}\!\varvec{\pm }\!{\textbf {0.04*}}$	0.83±0.07	0.57±0.02	0.56±0.03
		ANN01	2	0.65±0.05	0.72±0.03	0.52±0.09	0.83±0.03	0.81±0.09	0.52±0.03	0.51±0.03
		ANN02	3	0.65±0.01	0.74±0.03	0.56±0.06	0.84±0.04	0.83±0.07	0.56±0.02	0.52±0.04
	PWFP	KNN	2	0.56±0.04	0.54±0.04	0.60±0.08	0.52±0.05	0.77±0.03	0.43±0.03	0.44±0.03
		LDA	4	0.67±0.04	0.70±0.03	${\textbf {0.60}}\!\varvec{\pm }\!{\textbf {0.04}}$	0.74±0.05	0.82±0.03	0.53±0.06	0.50±0.04
		NB	4	${\textbf {0.68}}\!\varvec{\pm }\!{\textbf {0.05}}$	0.72±0.06	0.60±0.13	0.76±0.12	${\textbf {0.83}}\!\varvec{\pm }\!{\textbf {0.09}}$	0.54±0.03	0.51±0.04
		SVM	4	0.66±0.04	${\textbf {0.72}}\!\varvec{\pm }\!{\textbf {0.04}}$	0.52±0.07	${\textbf {0.80}}\!\varvec{\pm }\!{\textbf {0.06*}}$	0.81±0.09	0.51±0.02	0.50±0.03
		ANN01	3	0.61±0.05	0.69±0.06	0.51±0.05	0.76±0.07	0.78±0.07	0.47±0.05	0.48±0.04
		ANN02	4	0.64±0.03	0.71±0.03	0.55±0.03	0.78±0.04	0.81±0.04	${\textbf {0.55}}\!\varvec{\pm }\!{\textbf {0.03}}$	${\textbf {0.52}}\!\varvec{\pm }\!{\textbf {0.03}}$
	LNEC	KNN	3	0.60±0.04	0.60±0.04	0.58±0.08	0.61±0.06	0.79±0.04	0.45±0.03	0.43±0.04
		LDA	3	${\textbf {0.69}}\!\varvec{\pm }\!{\textbf {0.04*}}$	0.70±0.04	0.67±0.07	0.71±0.05	${\textbf {0.84}}\!\varvec{\pm }\!{\textbf {0.05*}}$	${\textbf {0.56}}\!\varvec{\pm }\!{\textbf {0.03*}}$	${\textbf {0.54}}\!\varvec{\pm }\!{\textbf {0.03*}}$
		NB	3	0.63±0.04	${\textbf {0.72}}\!\varvec{\pm }\!{\textbf {0.05*}}$	0.43±0.15	${\textbf {0.83}}\!\varvec{\pm }\!{\textbf {0.10*}}$	0.79±0.07	0.45±0.03	0.44±0.04
		SVM	4	0.58±0.13	0.56±0.18	0.62±0.17	0.54±0.28	0.75±0.13	0.45±0.08	0.43±0.05
		ANN01	3	0.56±0.01	0.69±0.03	${\textbf {0.69}}\!\varvec{\pm }\!{\textbf {0.04*}}$	0.72±0.04	0.78±0.03	0.52±0.04	0.50±0.04
		ANN02	3	0.66±0.01	0.62±0.06	0.61±0.09	0.60±0.06	0.73±0.07	0.43±0.06	0.41±0.05
WDBC	GFS	KNN	2	0.89±0.03	0.90±0.02	0.89±0.06	0.90±0.02	0.97±0.05	0.76±0.01	0.72±0.03
		LDA	2	0.93±0.02	${\textbf {0.95}}\!\varvec{\pm }\!{\textbf {0.01}}$	0.90±0.05	${\textbf {0.96}}\!\varvec{\pm }\!{\textbf {0.01*}}$	${\textbf {0.98}}\!\varvec{\pm }\!{\textbf {0.01}}$	${\textbf {0.87}}\!\varvec{\pm }\!{\textbf {0.03*}}$	${\textbf {0.83}}\!\varvec{\pm }\!{\textbf {0.05}}$
		NB	2	${\textbf {0.94}}\!\varvec{\pm }\!{\textbf {0.02}}$	0.94±0.01	${\textbf {0.94}}\!\varvec{\pm }\!{\textbf {0.03*}}$	0.94±0.02	${\textbf {0.98}}\!\varvec{\pm }\!{\textbf {0.01}}$	0.85±0.03	0.82±0.04
		SVM	2	0.92±0.02	0.92±0.01	0.91±0.05	0.93±0.02	${\textbf {0.98}}\!\varvec{\pm }\!{\textbf {0.01}}$	0.81±0.03	0.80±0.05
		ANN01	3	0.92±0.03	0.94±0.02	0.91±0.05	0.92±0.02	0.96±0.01	0.83±0.04	0.82±0.05
		ANN02	2	0.92±0.03	0.93±0.03	0.89±0.05	0.91±0.03	0.95±0.01	0.80±0.05	0.78±0.03
	PWFP	KNN	3	0.87±0.03	0.89±0.02	${\textbf {0.85}}\!\varvec{\pm }\!{\textbf {0.06}}$	0.90±0.03	0.96±0.06	0.74±0.01	0.72±0.02
		LDA	2	0.89±0.03	${\textbf {0.93}}\!\varvec{\pm }\!{\textbf {0.01}}$	0.83±0.06	${\textbf {0.95}}\!\varvec{\pm }\!{\textbf {0.01}}$	0.96±0.01	0.81±0.04	${\textbf {0.80}}\!\varvec{\pm }\!{\textbf {0.03}}$
		NB	2	0.89±0.03	0.92±0.01	${\textbf {0.85}}\!\varvec{\pm }\!{\textbf {0.06}}$	0.94±0.01	${\textbf {0.97}}\!\varvec{\pm }\!{\textbf {0.01}}$	${\textbf {0.81}}\!\varvec{\pm }\!{\textbf {0.03}}$	0.79±0.03
		SVM	1	0.86±0.03	0.88±0.02	0.84±0.06	0.88±0.02	0.96±0.02	0.71±0.05	0.68±0.04
		ANN01	4	0.85±0.01	0.88±0.02	0.82±0.05	0.89±0.02	0.95±0.01	0.79±0.04	0.75±0.03
		ANN02	2	${\textbf {0.90}}\!\varvec{\pm }\!{\textbf {0.01}}$	0.92±0.03	0.83±0.07	0.87±0.03	0.94±0.03	0.77±0.05	0.74±0.03
	LNEC	KNN	2	${\textbf {0.93}}\!\varvec{\pm }\!{\textbf {0.02}}$	${\textbf {0.94}}\!\varvec{\pm }\!{\textbf {0.01}}$	${\textbf {0.91}}\!\varvec{\pm }\!{\textbf {0.05}}$	0.94±0.02	${\textbf {0.98}}\!\varvec{\pm }\!{\textbf {0.01}}$	0.84±0.03	0.80±0.03
		LDA	3	0.92±0.03	0.93±0.01	0.89±0.05	0.94±0.01	0.97±0.01	0.83±0.04	0.81±0.04
		NB	1	0.90±0.03	0.94±0.02	0.84±0.06	${\textbf {0.97}}\!\varvec{\pm }\!{\textbf {0.02*}}$	0.96±0.01	0.84±0.05	0.81±0.02
		SVM	2	0.92±0.03	0.94±0.02	0.90±0.05	0.95±0.02	${\textbf {0.98}}\!\varvec{\pm }\!{\textbf {0.01}}$	0.85±0.04	${\textbf {0.83}}\!\varvec{\pm }\!{\textbf {0.03}}$
		ANN01	2	0.83±0.03	0.88±0.02	0.90±0.05	0.95±0.03	0.97±0.01	${\textbf {0.86}}\!\varvec{\pm }\!{\textbf {0.04}}$	0.82±0.02
		ANN02	3	0.91±0.02	0.92±0.02	0.89±0.05	0.93±0.02	0.96±0.01	0.82±0.04	0.79±0.03
GSE10810	GFS	KNN	1	0.95±0.05	0.96±0.04	0.98±0.04	0.92±0.10	0.97±0.07	0.97±0.03	${\textbf {0.96}}\!\varvec{\pm }\!{\textbf {0.02*}}$
		LDA	1	0.95±0.06	${\textbf {0.97}}\!\varvec{\pm }\!{\textbf {0.04}}$	${\textbf {1.00}}\!\varvec{\pm }\!{\textbf {0.00}}$	0.90±0.12	${\textbf {1.00}}\!\varvec{\pm }\!{\textbf {0.00}}$	${\textbf {0.98}}\!\varvec{\pm }\!{\textbf {0.03}}$	0.94±0.02
		NB	1	0.94±0.05	0.95±0.04	0.97±0.05	0.92±0.10	0.95±0.08	0.96±0.03	0.93±0.03
		SVM	2	${\textbf {0.96}}\!\varvec{\pm }\!{\textbf {0.05}}$	${\textbf {0.97}}\!\varvec{\pm }\!{\textbf {0.04}}$	0.99±0.03	0.92±0.10	0.99±0.04	${\textbf {0.98}}\!\varvec{\pm }\!{\textbf {0.03}}$	0.94±0.02
		ANN01	3	0.93±0.04	0.94±0.04	0.96±0.03	${\textbf {0.92}}\!\varvec{\pm }\!{\textbf {0.08}}$	0.96±0.04	0.97±0.03	0.94±0.02
		ANN02	3	0.95±0.06	0.93±0.05	0.95±0.03	0.90±0.13	0.95±0.05	0.94±0.04	0.92±0.03
	PWFP	KNN	3	0.70±0.11	0.65±0.12	0.52±0.16	${\textbf {0.88}}\!\varvec{\pm }\!{\textbf {0.14*}}$	0.51±0.10	0.64±0.15	0.60±0.10
		LDA	1	0.62±0.12	0.61±0.11	0.59±0.16	0.66±0.22	0.47±0.13	0.65±0.12	0.58±0.09
		NB	2	${\textbf {0.76}}\!\varvec{\pm }\!{\textbf {0.12*}}$	${\textbf {0.75}}\!\varvec{\pm }\!{\textbf {0.11*}}$	${\textbf {0.71}}\!\varvec{\pm }\!{\textbf {0.14*}}$	0.81±0.18	${\textbf {0.62}}\!\varvec{\pm }\!{\textbf {0.15*}}$	${\textbf {0.78}}\!\varvec{\pm }\!{\textbf {0.11*}}$	${\textbf {0.72}}\!\varvec{\pm }\!{\textbf {0.06*}}$
		SVM	3	0.61±0.10	0.56±0.11	0.43±0.17	0.79±0.18	0.44±0.09	0.54±0.14	0.56±0.12
		ANN01	3	0.65±0.06	0.67±0.04	0.57±0.03	0.77±0.08	0.58±0.09	0.66±0.13	0.61±0.10
		ANN02	3	0.66±0.05	0.64±0.05	0.51±0.08	0.74±0.12	0.56±0.11	0.62±0.12	0.60±0.11
	LNEC	KNN	3	0.62±0.13	0.61±0.12	0.59±0.12	0.65±0.21	0.47±0.12	0.66±0.11	0.63±0.10
		LDA	5	${\textbf {0.76}}\!\varvec{\pm }\!{\textbf {0.10*}}$	${\textbf {0.74}}\!\varvec{\pm }\!{\textbf {0.11*}}$	${\textbf {0.69}}\!\varvec{\pm }\!{\textbf {0.15*}}$	0.84±0.14	${\textbf {0.62}}\!\varvec{\pm }\!{\textbf {0.12*}}$	${\textbf {0.77}}\!\varvec{\pm }\!{\textbf {0.11*}}$	${\textbf {0.73}}\!\varvec{\pm }\!{\textbf {0.11*}}$
		NB	4	0.69±0.12	0.64±0.13	0.51±0.20	${\textbf {0.87}}\!\varvec{\pm }\!{\textbf {0.16}}$	0.52±0.14	0.62±0.18	0.61±0.13
		SVM	3	0.62±0.09	0.56±0.10	0.42±0.17	0.82±0.17	0.45±0.08	0.53±0.13	0.54±0.10
		ANN01	3	0.68±0.06	0.63±0.09	0.49±0.11	0.79±0.11	0.56±0.07	0.58±0.11	0.57±0.09
		ANN02	4	0.63±0.03	0.64±0.11	0.50±0.13	0.84±0.12	0.51±0.10	0.56±0.10	0.55±0.11
GSE15852	GFS	KNN	2	0.84±0.08	0.84±0.08	0.89±0.08	0.79±0.14	0.89±0.08	0.85±0.07	0.83±0.05
		LDA	3	0.83±0.08	0.83±0.08	${\textbf {0.98}}\!\varvec{\pm }\!{\textbf {0.05}}$	0.68±0.16	${\textbf {0.97}}\!\varvec{\pm }\!{\textbf {0.02}}$	0.85±0.06	0.82±0.05
		NB	4	${\textbf {0.89}}\!\varvec{\pm }\!{\textbf {0.07*}}$	${\textbf {0.89}}\!\varvec{\pm }\!{\textbf {0.07*}}$	0.96±0.07	${\textbf {0.81}}\!\varvec{\pm }\!{\textbf {0.13}}$	0.96±0.07	${\textbf {0.90}}\!\varvec{\pm }\!{\textbf {0.06}}$	${\textbf {0.88}}\!\varvec{\pm }\!{\textbf {0.06*}}$
		SVM	4	0.87±0.07	0.87±0.07	0.96±0.06	0.77±0.13	0.96±0.06	0.88±0.06	0.85±0.05
		ANN01	5	0.83±0.04	0.85±0.06	0.90±0.08	0.79±0.09	0.92±0.07	0.84±0.09	0.80±0.10
		ANN02	3	0.85±0.02	0.86±0.05	0.93±0.05	0.75±0.11	0.94±0.05	0.85±0.06	0.82±0.07
	PWFP	KNN	1	0.54±0.10	0.54±0.10	0.56±0.14	0.52±0.16	0.54±0.11	0.54±0.11	0.56±0.10
		LDA	1	0.59±0.11	0.59 ±0.11	0.65±0.15	0.54±0.16	0.61±0.12	0.61±0.11	0.58±0.11
		NB	3	0.57±0.11	0.57±0.11	0.70±0.17	0.44±0.15	0.62±0.18	0.61±0.12	0.59±0.10
		SVM	1	0.57±0.09	0.57±0.09	0.67±0.14	0.48±0.15	0.59±0.11	0.61±0.10	0.60±0.11
		ANN01	3	${\textbf {0.68}}\!\varvec{\pm }\!{\textbf {0.06*}}$	${\textbf {0.71}}\!\varvec{\pm }\!{\textbf {0.09*}}$	${\textbf {0.76}}\!\varvec{\pm }\!{\textbf {0.09*}}$	${\textbf {0.78}}\!\varvec{\pm }\!{\textbf {0.10*}}$	${\textbf {0.68}}\!\varvec{\pm }\!{\textbf {0.09*}}$	0.61±0.09	0.60±0.10
		ANN02	4	0.65±0.05	0.68±0.08	0.72±0.06	0.70±0.13	0.63±0.08	${\textbf {0.65}}\!\varvec{\pm }\!{\textbf {0.09*}}$	${\textbf {0.63}}\!\varvec{\pm }\!{\textbf {0.10*}}$
	LNEC	KNN	2	0.57±0.11	0.57±0.11	0.66±0.14	0.48±0.15	0.59±0.13	0.60±0.10	0.58±0.11
		LDA	4	${\textbf {0.61}}\!\varvec{\pm }\!{\textbf {0.12}}$	${\textbf {0.61}}\!\varvec{\pm }\!{\textbf {0.12*}}$	0.66±0.14	${\textbf {0.56}}\!\varvec{\pm }\!{\textbf {0.18}}$	${\textbf {0.63}}\!\varvec{\pm }\!{\textbf {0.15}}$	0.63±0.11	${\textbf {0.61}}\!\varvec{\pm }\!{\textbf {0.10}}$
		NB	2	0.57±0.10	0.57±0.10	${\textbf {0.73}}\!\varvec{\pm }\!{\textbf {0.15}}$	0.41±0.16	0.61±0.16	${\textbf {0.63}}\!\varvec{\pm }\!{\textbf {0.09}}$	0.59±0.10
		SVM	4	0.53±0.11	0.53±0.11	0.50±0.18	${\textbf {0.56}}\!\varvec{\pm }\!{\textbf {0.18}}$	0.53±0.11	0.50±0.14	0.48±0.12
		ANN01	2	0.60±0.01	0.58±0.11	0.70±0.08	0.52±0.10	0.55±0.10	0.61±0.13	0.58±0.11
		ANN02	3	0.57±0.09	0.59±0.07	0.65±0.16	0.52±0.14	0.60±0.09	0.59±0.11	0.53±0.10

Values in bold and with indicate higher mean values with significant difference *p*-value < 0.05), and values in bold denote relatively higher mean values or denote equal mean values with lower standard deviations

### Representative achievement on the BC datasets

Table 7 shows current achievement using FR/SFS and classifiers. On *BCDR-F03*, using 17 features [19] achieves 0.06 higher AUC over the present study. On *WDBC*, using 2 features in the present study achieves slightly lower AUC, ACC and SPE but higher SEN than that using 6 features with genetic algorithm [48]. On *GSE10810*, using 2 features from GFS leads to much better ACC over that using 80 features from the *t*-test in [49]. On *GSE15852*, using 4 features in the present study results in lower ACC than that using 235 features [50] and that using 10 features [51], while it achieves ACC close to that using 33 features [23] and to that using 50 features [52]. In general, GFS ranks lead to competitive or better performance as other FR/SFS methods when using much fewer features.

**Table 7:** Representative achievement on the BC datasets

		*n*	FR/SFS	Classifier	AUC	ACC	SEN	SPE
BCDR-F03	[19]	600		SVM	0.77±0.03			
	[19]	17		SVM	0.77±0.02			
	[53]	4	elastic net	SVM	0.69±0.05	0.74±0.05	0.56±0.10	0.81±0.08
	Ours	4	GFS	NB	0.71±0.04	0.77±0.03	0.59±0.10	0.84±0.05
WDBC	[54]	24	variable importance	hierarchical clustering RF	0.9896	0.9705	0.9477	0.9841
	[55]	14	genetic algorithm	particle swarm optimization		0.966	0.975	0.937
	[48]	6	genetic algorithm	kernel-based Bayesian	0.994	0.971	0.924	1.000
	[56]	14	genetic algorithm	rotation forest	0.993	0.9948		
	[57]	9	interaction dominance			0.9966		
	Ours	2	GFS	NB	0.94±0.02	0.94±0.01	0.94±0.03	0.94±0.02
GSE10810	[22]	8088	false discovery rate			1.000		
	[49]	80	*t*-test	SVM	0.7789			
	Ours	2	GFS	SVM	0.96±0.05	0.97±0.04	0.99±0.03	0.92±0.10
GSE15852	[23]	33	paired *t*-test	hierarchical cluster analysis		0.88	0.86	0.91
	[51]	10	logistic regression	RF		0.9311		
	[52]	50	prioritization analysis	SVM		0.87		
	Ours	4	GFS	NB	0.89±0.07	0.89±0.07	0.96±0.07	0.81±0.13

### Computational complexity analysis

The computational complexity of the proposed framework is from the FR/SFS algorithms ($\mathcal {O}_{fr}$), the stability estimator ($\mathcal {O}_{es}$) and the classifier ($\mathcal {O}_{class}$), which can be generally formulated as $\mathcal {O}_{fr} + \mathcal {O}_{es} + \mathcal {O}_{class}$.

In the proposed model, GFS is the FR algorithm, and $\mathcal {O}_{fr} = \mathcal {O}_{GFS} = \mathcal {O}(T(cns+s\log {m}))$ in which *T* is the number of iteration, *s* is the number of nonzero features among the training samples, *c* is the number of classes, *n* is the number of data samples, and *m* is the number of selected features [33]. The complexity of the estimator is $\mathcal {O}_{es} = \mathcal {O}(Mp)$ in which *M* is the number of feature sets and *p* is the feature dimensionality [17]. As to classifiers, the testing complexity of NB is $\mathcal {O}(cp)$ and that of linear SVM is $\mathcal {O}(p)$.

**Fig. 8:**
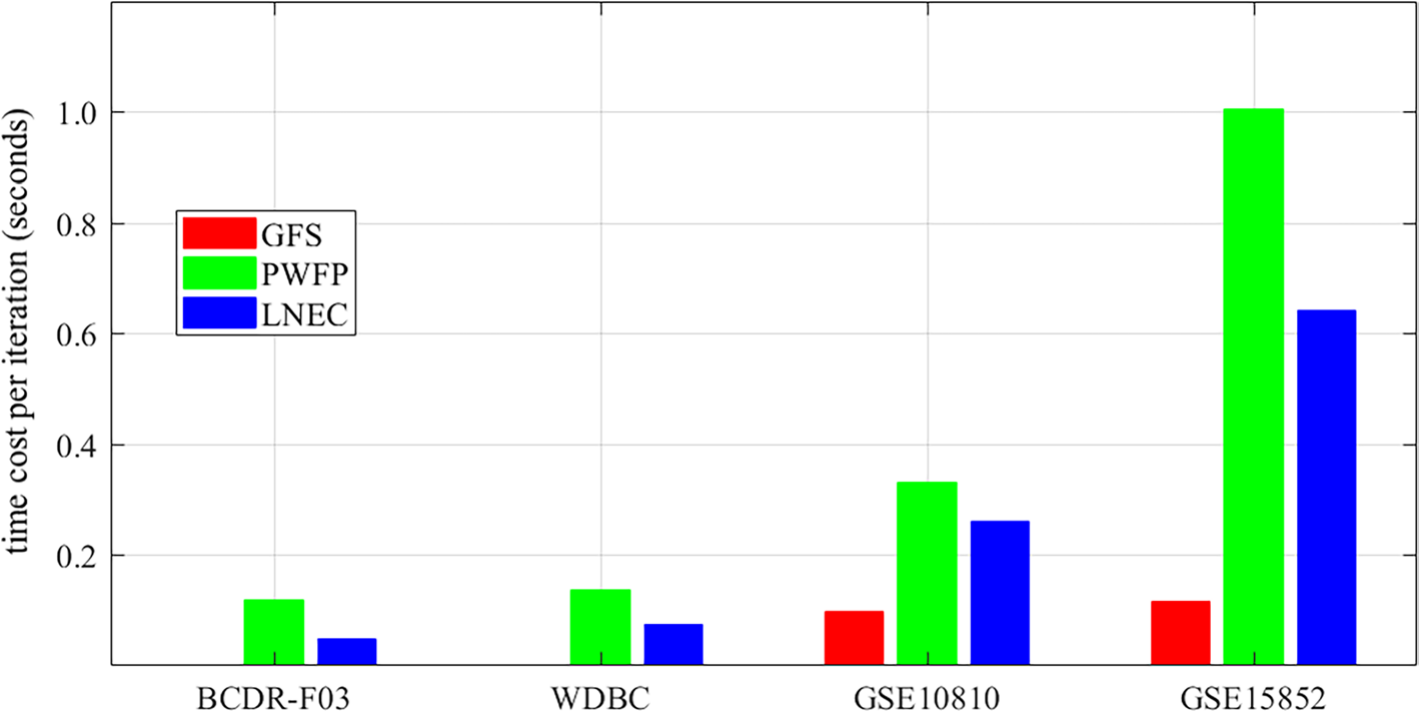
Average time cost per iteration for ranking features (GFS, red bar; PWFP, green bar; and LNEC, blue bar). (The figure can be enlarged for viewing)

Thus, the time cost of the proposed model is mainly laid on GFS algorithm. Figure 8 shows the time consumption for ranking features. It reveals that GFS is the fastest, and on GSE15852, its average time cost is $\approx$ 0.12 second per iteration.

## Discussion

A hybrid framework is proposed to identify stable FR algorithms for accurate BC diagnosis. Twenty-three algorithms have been evaluated on four datasets. It reveals that three algorithms show consistent stability, and GFS leads to superior prediction results.

Three algorithms show consistently good stability on the four datasets. Initially, 19 out of 42 algorithms handle GSE15852 [23] not well own to tens of thousands of gene features. Secondly, nearly all the remaining algorithms obtain stable feature ranks on BCDR-F03 and WDBC (Table 4), while substantially fewer algorithms show good stability on GSE10810 and GSE15850 (Table 5). The reason may come from the data sufficiency. It is easy to observe that there are more than eighteen samples to describe a feature on medical image dataset (BCDR-F03, 406 samples vs. 17 features; WDBC, 569 samples vs. 30 features), while on gene datasets, samples are far from sufficient (GSE10810, 58 samples vs. 18,382 genes; GSE15852, 86 samples vs. 22,283 genes) (Table 3). This finding might suggest that sufficient samples are necessitated for the construction of measure spaces before the estimation of feature importance [16]. Among the stable algorithms, GFS is the most efficient and it takes around 0.12 second to complete an iteration of the GSE15852 dataset (Fig. 8).

GFS ranks lead to superior diagnosis performance over the other two feature ranks (Table 6). On the medical image datasets, the evaluation metrics of GFS ranks show higher values over those of PWFP or LNEC rank with no significant difference (*p*-values > 0.05). For instance, GFS rank followed by KNN classifier (0.59±0.05) causes inferior SPE values in comparison to LNEC ranks with KNN classifier (0.61±0.06). On the gene expression datasets, GFS ranks result in significantly better performance over the other two ranks (*p*-values $< 10^{-8}$) regardless of classifiers. On another perspective, by using same classifiers, such as NB, on the datasets with sufficient samples (BCDR-F03 and WDBC), LNEC and PWFP ranks cause slightly inferior evaluation metric values in comparison to GFS ranks; while on gene expression datasets (GSE10810 and GSE15852), GFS ranks lead to much better results over the other two feature ranks. The comparison might reveal that GFS is able to discover signatures from high-dimensional small-sample gene datasets for improved BC diagnosis.

The proposed framework yields state-of-the-art performance (Table 7). On the gene expression datasets, using fewer gene features in this study exceeds some other methods on BC diagnosis. In [49], 80 genes cause inferior results on GSE10810. In [52], using 50 genes leads to worse performance on GSE15852. On the medical image datasets, using fewer features achieves comparable performance. On WDBC, 6 features lead to better result [48] than the present study using 2 features. On BCDR-F03, compared to the baseline work using 17 features [19], the present study using 4 features causes 0.06 AUC decrease. In general, using fewer features and simpler classifier in this study outperforms the other complex CAD models, such as hierarchical clustering RF [54] and particle swarm optimization [55], and the main contribution might come from the effective GFS feature ranks.

The selected features for accurate BC diagnosis have already been witnessed in previous studies or guidelines. On *BCDR-F03*, “circularity”, “perimeter” and “contrast” are found beneficial to breast image analysis. This finding is in consistent with the guideline of breast imaging-reporting and data system descriptor [58], and clinical studies identify that malignant lesions in MAM images are prone to show irregular shapes (“circularity” and “perimeter”) and inhomogeneous intensity (“contrast”). On *WDBC*, “concave points”, “the largest concave points” and “the largest perimeter” are vital in malignancy prediction. In an FNA image, “concave points” are the concave portions of the contour [20], and the presence of more concave points indicates a more irregular shape of a nucleus. The findings on BCDR-F03 and WDBC suggest that shape features should be paid more attention to MAM and FNA image analysis. On *GSE10810*, BC occurrence is highly related with genes “206930_at” and “222083_at” (both glycine-N-acyltransferase, GLYAT) and “243311_at” (defensin beta 132, DEFB132). Notably, the expression level of “206930_at”, “222083_at” and “243311_at” decrease from the normal (7.19±1.36, 7.59±1.07, and 7.12±1.81) to the tumor group (3.88±0.42, 4.89±0.43, and 4.22±0.40), with a significant difference (*p*-values $< 10^{-11}$). Existing studies have revealed that GLYAT-encoded proteins catalyze the transfer of acyl groups from acyl-CoA to glycine to produce acyl glycine and coenzyme A. The product acyl-CoA is an important resource for oxidative phosphorylation and lipogenesis that is necessary for normal cell metabolism. In particular, downregulation of GLYAT expression is associated with a variety of malignant tumors, including BC tumors [59]. DEFB132 is a member of the alarm element family. It mainly involves in the transmission of danger signals and may play a role in tumorigenesis [60]. On *GSE15852*, BC development is found in relation to genes “204997_at” (glycerol-3-phosphate dehydrogenase 1, GPD1), “210298_x_at” (four and a half LIM domains 1, FHL1), and “222317_at” (phosphodiesterase 3B, PDE3B). The expression level of the genes is significantly reduced from control cases to malignant cases (*p*-values $< 10^{-6}$). GPD1 encodes cytoplasmic NAD-dependent glycerol 3-phosphate dehydrogenase 1, a key element connecting carbohydrate and lipid metabolism. Existing studies have shown that GPD1 may inhibit the proliferation, migration, and invasion of breast cancer cells [61]. FHL1 has been identified as a suppressor gene for a variety of malignant tumors and exerts antitumor effects by inhibiting tumor differentiation, proliferation, invasion, and metastasis, and low FHL1 expression is closely related to the invasion and metastasis of breast cancer [62]. In addition, PDE3B-mediated cAMP hydrolysis limits the antiangiogenic potential of PKA in endothelial cells, suggesting PDE3B regulates angiogenesis and inhibits the occurrence and metastasis of breast cancer by controlling the invasion ability of endothelial cells [63].

In addition, selecting an appropriate classifier seems helpful when feature ranks are not so effective by comparing the classifiers. Taking LNEC ranks as an example, using LDA classifier generally obtains fair good results on the datasets (Table 6). In other words, using same feature subset from LNEC ranks, LDA generally outperforms the other classifiers in mapping features to the labels. It is also found that ANN with two hidden layers (ANN02) has no much improvement over that with one hidden layer (ANN01). The reason might come from the limited representation of quantitative features [19, 20] or the limited numbers of data samples [22, 23]. When feature ranks are fixed, which classifier is suitable for a specific task is a performance-oriented problem, which may require basic analysis, systematic experiments and empirical experience. Meanwhile, it is feasible to merge feature ranks into an optimization procedure for ensemble feature selection and malignancy prediction [64].

FR/SFS stability is crucial in cancer diagnosis, signature discovery and many other related applications. In the era of deep learning, FR/SFS stability provides a novel way to improve user confidence when deep networks are applied for high-risk decision-making tasks. It is known that deep networks can perform as feature extractors to generate massive hierarchical features [8, 12]. However, these features are so abstract that the decision-making procedures become uninterpretable. Alternatively, the stability or preference of deep features can be quantified as the frequency of features activated in the training stage [65], as the reproducibility of features when error rate is controlled via paired-input nonlinear knockoffs [66], or as the difference of propagating activation when decomposing the output prediction of a deep network based on a specific input of learned features [67]. Most importantly, FR/SFS stability should be considered before translating lab research findings to clinical practice, since only the features that have been stably identified as potential signatures deserve labor and time for further clinical investigation.

Several limitations exist in the current study. Firstly, on datasets with hundreds of samples, the impact of training size change on the stability estimation is an interesting topic. However, own to insufficient samples in gene datasets, the size of training samples is fixed. In our future study, the impact of training sizes will be explored. Secondly, using one estimator to assess the stability seems not convincing, while the estimator possesses all the properties of a good stability measure [17]. One desirable approach is to develop more estimators and to conduct comprehensive evaluation. Meanwhile, a decrease of the stability threshold can identify more FR algorithms, while it poses difficulty to follow-up data analysis, and thus, $S \ge 0.55$ is a trade-off. Thirdly, more advanced classifiers could be employed, such as deep learning networks [8], while to maintain good interpretability, six simple yet effective classifiers are applied. In our future work, more classifiers will be considered. On the other hand, instead of direct use of classifiers, one promising way is to embed feature ranks into an optimization procedure for signature discovery and cancer diagnosis [64]. Fourthly, retrieval and meta-analysis of discovered genes are helpful for understanding cancer occurrence, development and prognosis, while these topics fall outside of the scope of this study. In addition, using different data splitting strategies, such as k-fold cross validation and data percentage split criteria, might change the prediction results, while retaining the numbers of benign and malignant cases in the training set can avoid data imbalance and prediction bias. Last but not least, more efforts can be made to finely stratify patient cases from clinical data and cancer staging for personalized medicine.

## Conclusions

This study proposes a hybrid framework to investigate both the stability and effectiveness of FR algorithms on BC data analysis. Three algorithms exhibit good stability consistently on the datasets, and GFS feature ranks lead to superior classification performance. The GFS ranks suggest that shape features are vital in medical image analysis (BCDR-F03 and WDBC) and using a few of genes can help differentiation of benign and malignant cases (GSE10810 and GSE15852).

FR/SFS stability is important in real-world decision-making applications. This study indicates that few FR algorithms demonstrate stable feature preference on high-dimensional small-sample data analysis. To address this challenge, developing stable FR/SFS algorithms is preferred. Meanwhile, an effective reduction of feature dimensionality is also helpful for accurate estimation of feature importance. In addition, collecting sufficient samples is a primary consideration to determine the data distribution and to facilitate the stability estimation.

The proposed model could recognize stable FR/SFS algorithms and effective feature subsets. However, it is restricted to the input of quantitative features. The future scope of the model could be broadened into the deep learning field by concatenating low-, middle-, and high-level features of interest as the input. In the future, experiments will be conducted by involving more FR/SFS algorithms, machine learning classifiers, stability estimators and medical datasets for finding out stable and discriminative features for cancer diagnosis and signature discovery.

## Data Availability

The datasets BCDR-F03 (http://bcdr.inegi.up.pt), WDBC (https://archive.ics.uci.edu/ml/datasets/), GSE10810 (https://www.ncbi.nlm.nih.gov/geo/query/acc.cgi?acc=GSE10810), and GSE15852 (https://www.ncbi.nlm.nih.gov/geo/query/acc.cgi?acc=GSE15852) are all available online.

## Abbreviations

BC: Breast cancer
FR: Feature ranking
GFS: Generalized Fisher score
MAM: Mammography
FNA: Fine needle aspiration
CAD: Computer-aided diagnosis
FS: Feature selection
ANN: Artificial neural network
KNN: Knearest neighbors
LDA: Linear discriminant analysis
NB: Naive Bayes
RF: Random forest
SVM: Support vector machine
WDBC: Wisconsin diagnostic breast cancer
GEO: Gene Expression Omnibus
PWFP: Pairwise feature proximity
LNEC: L$_{2,0}$-norm equality constraints
AUC: Area under the receiver operating characteristic curve
ACC: Accuracy
SEN: Sensitivity
SPE: Specificity
MCC: Matthews correlation coefficient
NPV: Negative predictive value

## Footnotes

1
https://github.com/NicoYuCN/matFR

2
https://github.com/nogueirs/JMLR2018

## References

[1] Sung H, Ferlay J, Siegel RL, Laversanne M, Soerjomataram I, Jemal A (2021). Global cancer statistics 2020: GLOBOCAN estimates of incidence and mortality worldwide for 36 cancers in 185 countries. CA Cancer J Clin..

[2] Cao W, Chen HD, Yu YW, Li N, Chen WQ (2021). Changing profiles of cancer burden worldwide and in China: a secondary analysis of the global cancer statistics 2020. Chin Med J..

[3] Sharma R (2021). Global, regional, national burden of breast cancer in 185 countries: Evidence from GLOBOCAN 2018. Breast Cancer Res Treat..

[4] Barco I, Chabrera C, García-Fernández A, Fraile M, González S, Canales L (2017). Role of axillary ultrasound, magnetic resonance imaging, and ultrasound-guided fine-needle aspiration biopsy in the preoperative triage of breast cancer patients. Clin Transl Oncol..

[5] Reis-Filho JS, Pusztai L (2011). Gene expression profiling in breast cancer: classification, prognostication, and prediction. Lancet..

[6] Yu S, Wu S, Zhuang L, Wei X, Sak M, Neb D (2017). Efficient segmentation of a breast in B-mode ultrasound tomography using three-dimensional GrabCut (GC3D). Sensors..

[7] Houssein EH, Emam MM, Ali AA, Suganthan PN (2021). Deep and machine learning techniques for medical imaging-based breast cancer: A comprehensive review. Expert Syst Appl..

[8] Zou L, Yu S, Meng T, Zhang Z, Liang X, Xie Y. A technical review of convolutional neural network-based mammographic breast cancer diagnosis. Comput Math Methods Med. 2019;2019. Article ID 6509357. 10.1155/2019/6509357 PMC6452645 31019547

[9] Cai J, Luo J, Wang S, Yang S (2018). Feature selection in machine learning: A new perspective. Neurocomputing..

[10] Sun P, Wang D, Mok VC, Shi L (2019). Comparison of feature selection methods and machine learning classifiers for radiomics analysis in glioma grading. IEEE Access..

[11] Yu S, Liu L, Wang Z, Dai G, Xie Y (2019). Transferring deep neural networks for the differentiation of mammographic breast lesions. Sci China Technol Sci..

[12] Debelee TG, Schwenker F, Ibenthal A, Yohannes D (2020). Survey of deep learning in breast cancer image analysis. Evolving Syst..

[13] López NC, García-Ordás MT, Vitelli-Storelli F, Fernández-Navarro P, Palazuelos C, Alaiz-Rodríguez R (2021). Evaluation of feature selection techniques for breast cancer risk prediction. Int J Environ Res Public Health..

[14] Cueto-López N, García-Ordás MT, Dávila-Batista V, Moreno V, Aragonés N, Alaiz-Rodríguez R (2019). A comparative study on feature selection for a risk prediction model for colorectal cancer. Comput Methods Programs Biomed..

[15] Kalousis A, Prados J, Hilario M (2007). Stability of feature selection algorithms: a study on high-dimensional spaces. Knowl Inf Syst..

[16] Dernoncourt D, Hanczar B, Zucker JD (2014). Analysis of feature selection stability on high dimension and small sample data. Comput Stat Data Anal..

[17] Nogueira S, Sechidis K, Brown G (2017). On the stability of feature selection algorithms. J Mach Learn Res..

[18] Vakharia V, Gupta VK, Kankar PK (2016). A comparison of feature ranking techniques for fault diagnosis of ball bearing. Soft Comput..

[19] Arevalo J, González FA, Ramos-Pollán R, Oliveira JL, Lopez MAG (2016). Representation learning for mammography mass lesion classification with convolutional neural networks. Comput Methods Prog Biomed..

[20] Street WN, Wolberg WH, Mangasarian OL. Nuclear feature extraction for breast tumor diagnosis. In: Biomedical image processing and biomedical visualization. San Jose: SPIE; 1993; vol. 1905. p. 861–70.

[21] Edgar R, Domrachev M, Lash AE (2002). Gene Expression Omnibus: NCBI gene expression and hybridization array data repository. Nucleic Acids Res..

[22] Pedraza V, Gomez-Capilla JA, Escaramis G, Gomez C, Torné P, Rivera JM (2010). Gene expression signatures in breast cancer distinguish phenotype characteristics, histologic subtypes, and tumor invasiveness. Cancer Interdisc Int J Am Cancer Soc..

[23] Ni IBP, Zakaria Z, Muhammad R, Abdullah N, Ibrahim N, Emran NA (2010). Gene expression patterns distinguish breast carcinomas from normal breast tissues: the Malaysian context. Pathol-Res Pract..

[24] Zhang Z, Liang X, Qin W, Yu S, Xie Y (2020). matFR: a MATLAB toolbox for feature ranking. Bioinformatics..

[25] Cressie N, Whitford H (1986). How to use the two sample t-test. Biom J..

[26] Cover TM, Thomas JA (1991). Entropy, relative entropy and mutual information. Elem Inf Theory..

[27] Kailath T (1967). The divergence and Bhattacharyya distance measures in signal selection. IEEE Trans Commun Technol..

[28] Hsieh F, Turnbull BW (1996). Nonparametric and semiparametric estimation of the receiver operating characteristic curve. Ann Stat..

[29] Nachar N (2008). The Mann-Whitney U: A test for assessing whether two independent samples come from the same distribution. Tutor Quant Methods Psychol..

[30] Robnik-Šikonja M, Kononenko I (2003). Theoretical and empirical analysis of ReliefF and RReliefF. Mach Learn..

[31] Tibshirani R (1996). Regression shrinkage and selection via the lasso. J R Stat Soc Ser B (Methodol)..

[32] Roffo G. Feature selection library (MATLAB toolbox). 2016. arXiv preprint arXiv:1607.01327.

[33] Gu Q, Li Z, Han J. Generalized fisher score for feature selection. 2012. arXiv preprint arXiv:1202.3725.

[34] Uitdehaag J, Zaman GJ (2011). A theoretical entropy score as a single value to express inhibitor selectivity. BMC Bioinformatics..

[35] McKight PE, Najab J. Kruskal-wallis test. Corsini Encycl Psychol. 2010;1.

[36] Happy S, Mohanty R, Routray A, An effective feature selection method based on pair-wise feature proximity for high dimensional low sample size data. In: 2017 25th European signal processing conference (EUSIPCO). Kos Island: IEEE; 2017. p. 1574–8.

[37] Hu W, Choi KS, Gu Y, Wang S (2013). Minimum-maximum local structure information for feature selection. Pattern Recogn Lett..

[38] Zeng H, Cheung Y-M (2010). Feature selection and kernel learning for local learning-based clustering. IEEE Trans Pattern Anal Mach Intell..

[39] Roffo G, Melzi S. Features selection via eigenvector centrality. In: Proceedings of new frontiers in mining complex patterns (NFMCP 2016) (Oct 2016). Riva del Garda: Springer International Publishing; 2016.

[40] Roffo G, Melzi S, Castellani U, Vinciarelli A. Infinite latent feature selection: A probabilistic latent graph-based ranking approach. In: Proceedings of the IEEE international conference on computer vision. Santiago: IEEE; 2017. p. 1398–1406.

[41] Bradley PS, Mangasarian OL. Feature selection via concave minimization and support vector machines. In: ICML. 1998;98:82–90.

[42] Roffo G, Melzi S, Cristani M. Infinite feature selection. In: Proceedings of the IEEE International Conference on Computer Vision. 2015. p. 4202–10.

[43] He X, Cai D, Niyogi P. Laplacian score for feature selection. Adv Neural Inf Process Syst. 2005;18.

[44] Guo J, Zhu W. Dependence guided unsupervised feature selection. In: Proceedings of the AAAI Conference on Artificial Intelligence. Louisiana: AAAI; 2018. vol. 32.

[45] Du L, Shen YD. Unsupervised feature selection with adaptive structure learning. In: Proceedings of the 21th ACM SIGKDD international conference on knowledge discovery and data mining. Sydney: ACM; 2015. p. 209–18.

[46] Shi L, Du L, Shen YD. Robust spectral learning for unsupervised feature selection. In: 2014 IEEE International Conference on Data Mining. Shenzhen: IEEE; 2014. p. 977–982.

[47] Qian M, Zhai C. Robust unsupervised feature selection. In: Twenty-third international joint conference on artificial intelligence. Beijing: Morgan Kaufmann; 2013.

[48] Wuniri Q, Huangfu W, Liu Y, Lin X, Liu L, Yu Z (2019). A generic-driven wrapper embedded with feature-type-aware hybrid Bayesian classifier for breast cancer classification. IEEE Access..

[49] Zheng F, Wei L, Zhao L, Ni F. Pathway network analysis of complex diseases based on multiple biological networks. BioMed Res Int. 2018;2018. Article ID 5670210. 10.1155/2018/5670210 PMC6091292 30151386

[50] Jia D, Chen C, Chen C, Chen F, Zhang N, Yan Z (2021). Breast cancer case identification based on deep learning and bioinformatics analysis. Front Genet..

[51] Sun M, Ding T, Tang XQ, Yu K (2018). An efficient mixed-model for screening differentially expressed genes of breast cancer based on LR-RF. IEEE/ACM Trans Comput Biol Bioinform..

[52] Zhang Y, Li W, Zhang Y, Hu E, Rong Z, Ge L (2020). Network-based integration method for potential breast cancer gene identification. J Cell Physiol..

[53] Yu S, Chen H, Yu H, Zhang Z, Liang X, Qin W, et al. Elastic Net based Feature Ranking and Selection. 2020. arXiv preprint arXiv:2012.14982.

[54] Huang Z, Chen D (2021). A breast cancer diagnosis method based on VIM feature selection and hierarchical clustering random forest algorithm. IEEE Access..

[55] Aalaei S, Shahraki H, Rowhanimanesh A, Eslami S (2016). Feature selection using genetic algorithm for breast cancer diagnosis: experiment on three different datasets. Iran J Basic Med Sci..

[56] Aličković E, Subasi A (2017). Breast cancer diagnosis using GA feature selection and Rotation Forest. Neural Comput Appl..

[57] Zeng Z, Heng X. Feature selection and visualization based on interaction dominance. In: 2019 IEEE Fourth International Conference on Data Science in Cyberspace (DSC). Hangzhou: IEEE; 2019. p. 668–73.

[58] Spak DA, Plaxco J, Santiago L, Dryden M, Dogan B (2017). BI-RADS® fifth edition: A summary of changes. Diagn Interv Imaging..

[59] Tian X, Wu L, Jiang M, Zhang Z, Wu R, Miao J (2021). Downregulation of GLYAT Facilitates Tumor Growth and Metastasis and Poor Clinical Outcomes Through the PI3K/AKT/Snail Pathway in Human Breast Cancer. Front Oncol..

[60] Coffelt SB, Scandurro AB (2008). Tumors sound the alarmin (s). Cancer Res..

[61] Zhou C, Yu J, Wang M, Yang J, Xiong H, Huang H (2017). Identification of glycerol-3-phosphate dehydrogenase 1 as a tumour suppressor in human breast cancer. Oncotarget..

[62] Li Y, Qiu J, Pang T, Ye F, Huang L, Zhang X (2020). MiR-183-5p promotes proliferation, metastasis and angiogenesis in breast cancer cells through negatively regulating four and a half LIM protein 1. J Breast Cancer..

[63] MacKeil JL, Brzezinska P, Burke-Kleinman J, Theilmann AL, Nicol CJ, Ormiston ML (2019). Phosphodiesterase 3B (PDE3B) antagonizes the anti-angiogenic actions of PKA in human and murine endothelial cells. Cell Signal..

[64] Bolón-Canedo V, Alonso-Betanzos A (2019). Ensembles for feature selection: A review and future trends. Inf Fusion..

[65] Antropova N, Huynh BQ, Giger ML (2017). A deep feature fusion methodology for breast cancer diagnosis demonstrated on three imaging modality datasets. Med Phys..

[66] Lu Y, Fan Y, Lv J, Stafford Noble W. DeepPINK: reproducible feature selection in deep neural networks. Adv Neural Inf Processing Syst. 2018;31.

[67] Shrikumar A, Greenside P, Kundaje A. Learning important features through propagating activation differences. In: International conference on machine learning. PMLR. 2017;70:3145–53.

